# Clinical Neuropathology image 6-2018: Metastasis of breast carcinoma to meningioma 

**DOI:** 10.5414/NP301150

**Published:** 2018-10-22

**Authors:** Sigrid Klotz, Christian Matula, Matthias Pones, Merima Herac, Anna Grisold, Johannes A. Hainfellner, Gabor G. Kovacs, Ellen Gelpi

**Affiliations:** 1 Institute of Neurology, Medical University of Vienna, Vienna,; 2 Department of Neurosurgery, Medical University of Vienna, Vienna,; 3 Department of Radiology, Medical University of Vienna, Vienna,; 4 Institute of Pathology, and; 5 Department of Neurology, Medical University of Vienna, Vienna, Austria

**Keywords:** meningioma, metastasis, GATA3, breast cancer

## Abstract

No abstract available.

We report a case of a 33-year-old woman with history of stage IV breast cancer diagnosed at the age of 30 with metastasis to a meningioma. She presented with progressive headache and a gradual reduction of vision over the course of 10 days. MRI revealed multiple intracranial lesions suspicious of metastases. In two of them, the differential diagnosis with meningiomas was raised. One of the latter tumors compressed the optical nerves (right > left) and the optic chiasm, which indicated immediate microsurgical excision of the tumor. Neuropathological examination revealed two intimately connected neoplastic lesions. One lesion showed features of a meningotheliomatous meningioma, and within it, large epithelial, highly proliferating tumor nests were identified (Figure 1A). The epithelial nests showed strong nuclear immunoreactivity for GATA3 (Figure 1C), supporting an origin from the primary breast carcinoma [1]. In contrast, meningioma cells showed prominent nuclear immunoreaction for progesterone receptor (Figure 1B) and remained negative for GATA3. The diagnosis of a metastasis of a breast carcinoma to a meningotheliomatous meningioma was made. 

Tumor-to-tumor metastasis has been described before, although it presents a rather uncommon occurrence [1, 2]. Meningiomas are the most common intracranial recipient tumors of tumor-to-tumor metastasis [2, 3]. Breast and lung carcinomas are the most common origin of metastases to other tumors [1, 2, 4]. 

Different hypotheses to the pathogenesis of malignomas metastasizing into meningiomas have been proposed. First, a high percentage of meningiomas express progesterone receptors, as seen in the present case (Figure 1A), which could be a contributing factor to metastasis formation of breast cancer in meningioma [5]. In addition, the rich vascularization and the drainage by the dural sinus could facilitate hematogenous spread into the meningioma [1]. Moreover, the meningioma might provide an ideal environment for other tumors to grow in, not only because of an ideal nutritional microenvironment, but also because of an altered immunological condition and a non-competitive setting due to the slow growth of the meningioma [4, 6]. And finally, the observation of a metastasis of a pulmonary carcinoma in the same localization where a meningioma had been removed 11 months earlier suggests that there might be other advantageous factors in the surroundings of meningiomas [7]. 

This case presents a special constellation of a patient with two different rare occurrences: a young age for suffering from breast cancer and the metastasis to a meningioma. Whether there is a common link between these two situations remains unclear, as well as the exact mechanisms involved in tumor-to-tumor metastasis. Further studies are needed to better characterize this phenomenon. 

## Funding 

There was no specific funding for this study. 

## Conflict of interest 

The authors declare no conflict of interest. 

**Figure 1.**
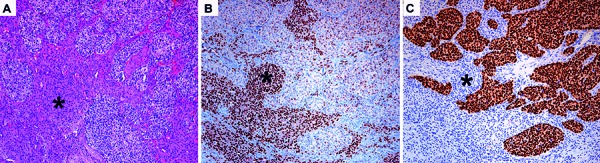
A: Hematoxylin & Eosin stained section shows the two different neoplastic components. On the lower left part of the figure, parts of a meningioma with formation of whorls (asterisk), on the upper right part of the figure nests of an epithelial neoplasm. B: Strong nuclear immunoreactivity for progesterone-receptor in the meningioma (asterisk). C: Strong nuclear immunoreactivity of the metastasis for GATA3. Note the negative staining in the meningioma (asterisk and surroundings). All images magnification × 100.

## References

[1] Conzen M Sollmann H Schnabel R Metastasis of lung carcinoma to intracranial meningioma. Case report and review of literature. Neurochirurgia (Stuttg). 1986; 29: 206–209. 3785498 10.1055/s-2008-1054162

[2] Takei H Powell SZ Tumor-to-tumor metastasis to the central nervous system. Neuropathology. 2009; 29: 303–308. 18647266 10.1111/j.1440-1789.2008.00952.x

[3] Das S Chaudhary N Ang L-C Megyesi JS Papillary thyroid carcinoma metastasizing to anaplastic meningioma: an unusual case of tumor-to-tumor metastasis. Brain Tumor Pathol. 2017; 34: 130–134. 28600666 10.1007/s10014-017-0289-5

[4] Richter B Harinath L Pu C Stabingas K Metastatic spread of systemic neoplasms to central nervous system tumors: review of the literature and case presentation of esophageal carcinoma metastatic to meningioma. Clin Neuropathol. 2017; 36: 60–65. 28025960 10.5414/NP300980

[5] Iplikcioglu AC Hatiboglu MA Ozek E Ozcan D Is progesteron receptor status really a prognostic factor for intracranial meningiomas? Clin Neurol Neurosurg. 2014; 124: 119–122. 25036873 10.1016/j.clineuro.2014.06.015

[6] Lanotte M Benech F Panciani PP Cassoni P Ducati A Systemic cancer metastasis in a meningioma: report of two cases and review of the literature. Clin Neurol Neurosurg. 2009; 111: 87–93. 18930586 10.1016/j.clineuro.2008.07.011

[7] el Sharouni SY Berfelo MW Theunissen PH Jager JJ de Jong JM A unique case of intracranial metastasis from lung carcinoma. Clin Neurol Neurosurg. 1993; 95: 257–260. 8242972 10.1016/0303-8467(93)90134-3

